# An Adaptive Actuation Mechanism for Anthropomorphic Robot Hands

**DOI:** 10.3389/frobt.2019.00047

**Published:** 2019-07-05

**Authors:** George P. Kontoudis, Minas Liarokapis, Kyriakos G. Vamvoudakis, Tomonari Furukawa

**Affiliations:** ^1^Department of Mechanical Engineering, Virginia Tech, Blacksburg, VA, United States; ^2^Department of Mechanical Engineering, University of Auckland, Auckland, New Zealand; ^3^Daniel Guggenheim School of Aerospace Engineering, Georgia Institute of Technology, Atlanta, GA, United States

**Keywords:** bioinspiration, underactuation, tendon-driven mechanisms, robotic fingers, robot hands

## Abstract

This paper presents an adaptive actuation mechanism that can be employed for the development of anthropomorphic, dexterous robot hands. The tendon-driven actuation mechanism achieves both flexion/extension and adduction/abduction on the finger's metacarpophalangeal joint using two actuators. Moment arm pulleys are employed to drive the tendon laterally and achieve a simultaneous execution of abduction and flexion motion. Particular emphasis has been given to the modeling and analysis of the actuation mechanism. More specifically, the analysis determines specific values for the design parameters for desired abduction angles. Also, a model for spatial motion is provided that relates the actuation modes with the finger motions. A static balance analysis is performed for the computation of the tendon force at each joint. A model is employed for the computation of the stiffness of the rotational flexure joints. The proposed mechanism has been designed and fabricated with the hybrid deposition manufacturing technique. The efficiency of the mechanism has been validated with experiments that include the assessment of the role of friction, the computation of the reachable workspace, the assessment of the force exertion capabilities, the demonstration of the feasible motions, and the evaluation of the grasping and manipulation capabilities. An anthropomorphic robot hand equipped with the proposed actuation mechanism was also fabricated to evaluate its performance. The proposed mechanism facilitates the collaboration of actuators to increase the exerted forces, improving hand dexterity and allowing the execution of dexterous manipulation tasks.

## 1. Introduction

The fields of robot grasping and dexterous manipulation have received increased attention over the last years, as robots have already started to interact with their surroundings and assist humans in the execution of dexterous tasks. Since the human hand is considered to be Nature's most dexterous end-effector, the prospect of replicating human dexterity has motivated roboticists to follow bio-inspired approaches (Dalley et al., 2009; Deshpande et al., 2013; Xiong et al., 2016; Xu and Todorov, 2016). One of the most important joints in the human hand is the metacarpophalangeal joint (MCP), which allows the fingers to execute both adduction/abduction and flexion/extension motions, thus increasing the dexterity of the overall system. Moreover, the human thumb's MCP joint along with the trapeziometacarpal and interphalangeal joints are responsible for opposition, which is the most significant motion that contributes to the dexterity of human hand (Kapandji, 1974; Nanayakkara et al., 2017).

The goal of this work is to enhance the robotic finger's performance in order to facilitate the execution of various grasping and in-hand manipulation tasks by employing less actuators without compromising dexterity, as discussed in (Bicchi, 2000). To this end, we propose a versatile, tendon-driven actuation mechanism for anthropomorphic fingers. These fingers are considered adaptive, since they are underactuated and they use structural compliance. The main contribution is the tendon-driven actuation mechanism. The design is minimal and modular, while it makes use of simple mechanical elements. We investigate the performance of adaptive fingers that employ flexure joints based on elastomer material (urethane rubber). The proposed mechanism has the ability to perform concurrent flexion/extension and adduction/abduction on the MCP joint by employing two actuators. We present a modeling framework to compensate for gravity with a torsional spring. We also provide design parameters for various adduction/abduction motions through a mechanism analysis. A model of the finger in spatial motion execution is discussed. We employ the smooth curvature model to analytically compute the stiffness of each rotational flexure joint. The mechanism improves the reachable workspace and amplifies the exerted finger forces. It is to be noted that the proposed mechanism can be used in other applications (e.g., development of a bio-inspired shoulder).

The remainder of this paper is organized as follows. Section 2 surveys the related work. Section 3 focuses on the design of the actuation mechanism and performs an analysis of the design constraints. Section 4 describes the fabrication process. Section 5 presents the results of the simulations and the experimental validation. Section 6 presents limitations of the proposed mechanism, while section 7 concludes the paper and discusses future directions. A numerical example of the adaptive finger characteristics is provided in the Appendix (Supplementary Material).

## 2. Related Work

Traditionally, the problems of robot grasping and dexterous manipulation have been addressed using rigid, fully actuated, multi-fingered robot hands (Lovchik and Diftler, 1999; Butterfaß et al., 2001; Kawasaki et al., 2002). These devices are typically heavy, expensive, difficult to build and maintain, and they rely on sophisticated sensing elements and complicated control laws in order to operate in unstructured and dynamic environments. The Awiwi hand Grebenstein (2012), equips the DLR hand arm system (Grebenstein et al., 2011). The hand consists of 38 motors for the actuation of 19 degrees of freedom (DoF). Each DoF has an antagonistic actuation scheme, being able to exert forces even during the extension of the fingers. The actuation is tendon-driven and each tendon contributes to a particular motion. Furthermore, the hand is comprised of multiple sensing elements that allow for precise grasping and in-hand manipulation. However, the hand is heavyweight, requires sophisticated control laws (just the actuation space is of 38 dimension), the fabrication process is complex, and its cost is significantly high.

Recently, a new class of adaptive robot hands was introduced (Dollar and Howe, 2010; Aukes et al., 2014; Catalano et al., 2014; Ciocarlie et al., 2014; Kontoudis et al., 2015; Deimel and Brock, 2016), that attempts to revolutionize the fields of robot grasping and manipulation, by simplifying the extraction of robust grasps under object pose uncertainties and the execution of dexterous, in-hand manipulation tasks. These hands are considered adaptive, since they are equipped with flexure or spring loaded pin joints, underactuated fingers, and differential mechanisms. Differential mechanisms allow the hands to conform to unknown/arbitrary object shapes without any means of feedback.

Although adaptive hands have been extensively used for robust grasping, only few studies have employed them for complex, in-hand manipulation tasks. The EthoHand (Konnaris et al., 2016), an anthropomorphic, tendon-driven robot hand, is actuated by seven motors and achieves in-hand object rolling. The actuation scheme results in an actuators-to-fingers ratio of 1.4, from which three are used for the ball joint of the thumb and the rest of the actuators for the four fingers^1^. In the thumb, the three tendon routing systems contribute to separate motions. One tendon routing system is responsible for the flexion/extension, one for the adduction, and one for the abduction. The four fingers perform only flexion/extension, without any adduction/abduction. Particularly, lateral motion of the four fingers is not considered in the design. In Odhner et al. (2014), the authors proposed the i-HY, an adaptive robot hand that was developed specifically for dexterous manipulation. The authors achieved dexterous finger interdigitation and finger pivoting by employing five actuators to control three fingers. Two actuators were dedicated explicitly to adduction/abduction without any contribution to the force exertion capabilities of the flexion/extension. This leads to an actuators-to-fingers ratio of 1.66. The Pisa/IIT SoftHand 2 (Della Santina et al., 2018), is an anthropomorphic, adaptive robot hand that employs two motors, having an actuators-to-fingers ratio of 0.4. The authors used a single tendon routing system without differential mechanisms. The hand relies in postural synergies for grasping (Santello et al., 1998), postural synergies during grasping by exploiting environmental constraints (Della Santina et al., 2017), and postural synergies during manipulation (Todorov and Ghahramani, 2004). In terms of motion skills, the hand is able to perform asynchronous active flexion, passive extension, and passive adduction/abduction. In terms of in-hand manipulation capabilities, the hand achieves object rolling of various objects with a fixed equilibrium point.

In Ryew and Choi (2001), the authors proposed a double active universal joint (DAUJ) that was implemented with gear transmission and two actuators. Their focus was on in-pipe inspection systems and robotic fingers with pin joints. In the work of Lotti et al. (2005), the authors presented the UBH 3, which was equipped with tendon-driven, spring loaded pin joints on fingers. The robot hand was able to perform grasping and in-hand manipulation using 16 actuators. The authors in Xu et al. (2012) introduced an anthropomorphic robotic finger with pin joints that employed biomimetic crocheted ligaments and a tendon-routing system. Their objective was to develop a robotic finger that has an identical structure as that of human fingers. In Kuo and Deshpande (2016), the authors proposed a compliant robotic finger design that integrates passive parallel compliance. Their design combines elastomer materials along with a specific structure that performs as a variable stiffness compliant joint toward improving the stability in grasping and manipulation. In Scarcia et al. (2017), the authors presented a rotational elastic joint for underactuated robotic fingers. Their design is monolithic and the joint is implemented with an embedded spiral torsional spring. The flexion/extension analysis for robotic fingers with pin joints and flexure joints has been studied in Birglen and Gosselin (2004), Odhner and Dollar (2012), and Niehues et al. (2017), respectively. In Hussain et al. (2018) an analytical modeling of flexure joints based on screw theory was presented. In addition, the authors fabricated a gripper to demonstrate its grasping capabilities.

Monolithic structures can significantly simplify the manufacturing process and reduce the manufacturing cost, as they require a single step process (Kota et al., 2001). They also lack wear, backlash, and friction, which impacts to minimal detrimental effect (Howell, 2012). In the work of Ananthasuresh et al. (1994), the authors presented a methodology to design compliant mechanisms based on the topology optimization homogenization method (Bendsøe and Kikuchi, 1988). However, when the employed material is flexible, then the rigid body assumption is not guaranteed for all geometries. A soft monolithic finger was presented in Mutlu et al. (2016). The authors investigated various types of flexure joints and fabricated an adaptive finger. Yet, the adduction/abduction motion was not studied.

Regarding the anthropomorphism of robot motion, in a previous study, Liarokapis et al. (2013) investigated the affinity in structure and motion of robotic hands and the human hand. The human hand is compared in two different stages with various robot hands by employing computational geometry and set theory methods to derive a comprehensive index of anthropomorphism that can be used for design optimization purposes.

## 3. Finger Design and Actuation Mechanism

In this section, we present the design of a versatile adaptive finger and we describe a tendon-driven actuation mechanism that allows the finger to perform both flexion/extension and adduction/abduction. Next, we present the modeling framework to compensate for gravity with mechanical elements. The actuation mechanism analysis is provided to specify design parameters for various applications. Then, the adaptive finger modeling for spatial motions is analyzed.

### 3.1. Adaptive Finger and Actuation Mechanism Design

**Figure 1:**
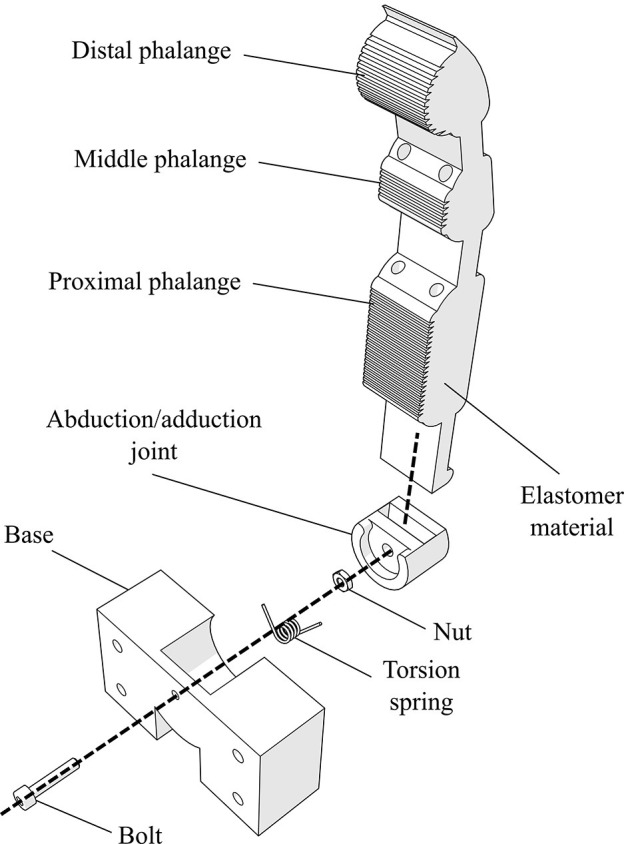
An exploded view of the finger's 3D model design. The finger structure is compliant as it combines an elastic body (urethane rubber) with plastic parts (e.g., tendon routing tubes and joint base). The design is also modular, as it is connected to the palm with a single bolt-nut set.

The finger structure is monolithic and consists of an elastic body (made out of urethane rubber) and plastic parts, as presented in Figure 1. The robotic finger is actuated by artificial tendons and their structure is presented with dashed lines. The distal, middle, and proximal phalanges as well as the flexure joints (areas of reduced thickness) are implemented with an elastomer material. The MCP spring loaded pin joint is responsible for the adduction/abduction. We target this motion because it enhances the grasping capabilities of robot hands as presented in Santello et al. (1998). More specifically, the abduction is the dominant motion of the second principal component that augments the grasping capabilities by 17%. Also, according to Odhner et al. (2014), finger abduction improves the dexterity of in-hand manipulation capabilities. The monolithic structure from flexible materials adds compliance to the design. Besides the MCP joint's motion, the design is also modular since the fingers are attached to the base frame (palm) with a single bolt-nut set.

**Figure 2:**
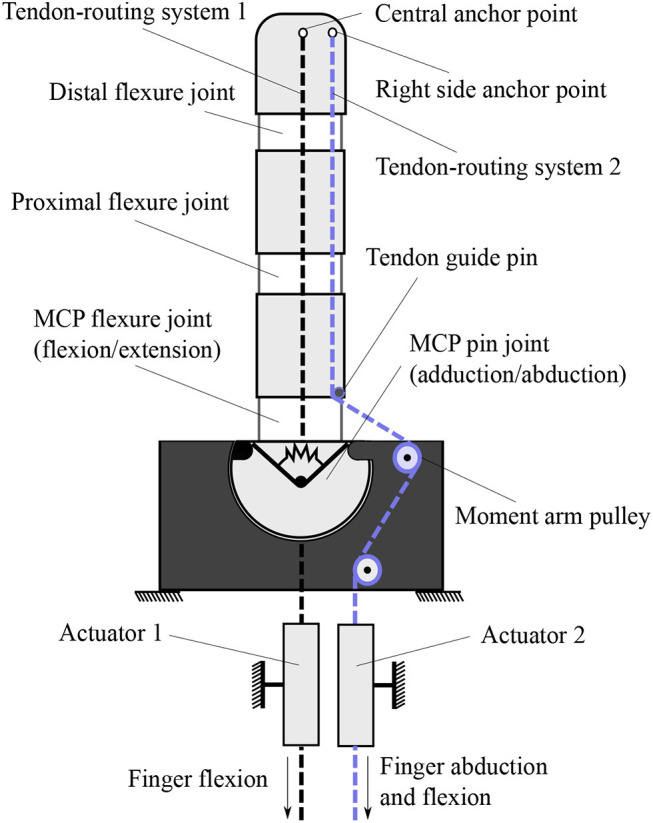
The actuation mechanism that allows for flexion/extension and adduction/abduction concurrently. This finger operates a clockwise motion. For counterclockwise motion, the right side anchor point needs to be swifted on the left side. For bidirectional abduction, the central anchor point needs to be placed on the left side of the finger.

The actuation mechanism utilizes two independent tendon routing systems to actuate the finger, as shown in Figure 2. We equip the proposed actuation mechanism with moment arm pulleys to drive the tendon routing system through a specific path, which is illustrated with the dashed blue line. On this path, the line of action of the applied force is increased. Therefore, the forces transferred through the tendon routing system create a moment that rotates the finger. Each tendon is responsible for a different motion. The tendon with an ending point at the central anchor (connected with the first actuator) is only responsible for the flexion/extension motion of the finger. The tendon with an ending point at the right side anchor point (connected with the second actuator) triggers initially the adduction/abduction motion and then contributes to the flexion/extension of the finger. This two-fold contribution in motion and force transmission lies in the design choice to place the right side anchor point at the distal phalange and not to the middle or the proximal phalange. In case of concurrent actuation of both motors the flexion/extension is the dominant motion.

The selection of anchor points for each separate tendon routing system is determined according to the desired finger motion. One can notice that from the human hand's neutral position, the index abduction moves oppositely from the ring and pinky abduction motions. The abduction motion from the natural position of the middle finger can be neglected since it is relatively small. On the other hand, the thumb motion includes bidirectional adduction/abduction. Therefore, for an anthropomorphic hand design we should be able to produce three different types of finger abductions. For this purpose, we employ right-side anchor points for clockwise motion, left-side anchor points for counterclockwise motion, and both-sides anchor points for bidirectional rotation. In case that we pursue single side rotation, the utilization of central anchor points is imposed to actuate the finger flexion/extension movements. It must be noted that for bidirectional adduction/abduction the termination point is crucial. In particular, in case the termination of the tendon routing system is placed in any other phalange than the distal, the flexion/extension will not be feasible.

### 3.2. Finger Rigid Body Modeling

**Figure 3:**
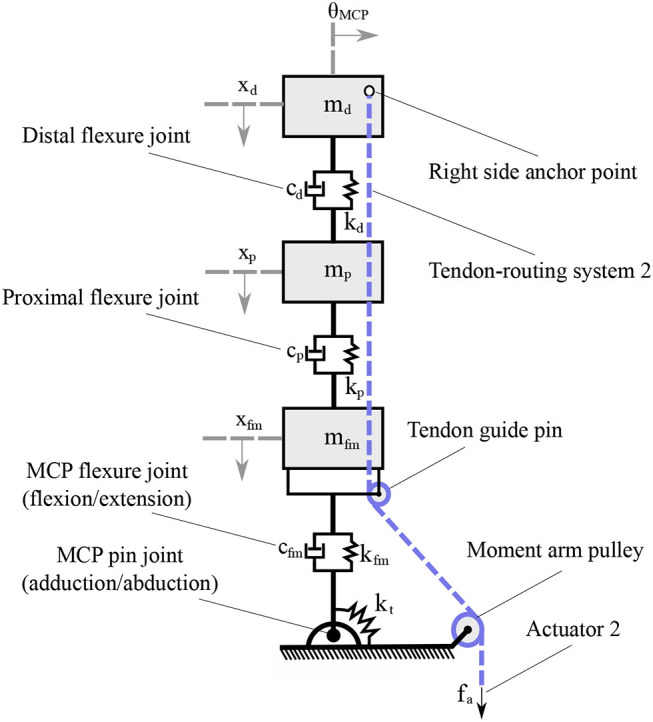
The mechanical model of a compliant finger that achieves a clockwise rotation. The flexure joints were modeled as a spring-damper and each phalange as a mass. The flexion/extension of the DIP, PIP, and MCP joints reflects the *x*_d_, *x*_p_, and *x*_fm_ motions, respectively, while the adduction/abduction occurs toward the θ rotational direction.

In Figure 3, a mechanical model of the compliant robotic finger is presented. We employ a spring-mass system to model the finger and its compliant flexure joints. When no contact occurs and since the finger makes use of two tendon routing systems, it eliminates by design the out-of-plane motions of the elastomer material such as twisting and lateral bending. A similar flexure joint modeling is discussed in Kim et al. (2005). The mechanical model consists of discrete mass nodes distributed throughout the finger. The masses *m*_d_, *m*_m_, *m*_p_ represent the distal, the middle, and the proximal phalange masses respectively. The stiffness *k*_d_, *k*_p_, *k*_fm_ correspond to the distal interphalangeal (DIP), the proximal interphalangeal (PIP), and the MCP flexure joints, respectively. The spring with stiffness *k*_t_ models the torsional spring. The dashed line is the tendon that triggers first the adduction/abduction and then contributes to the flexion/extension motion. The force *f*_a_, at the end of the tendon routing system is the actuator force. It must be noted that we refer to linear displacement spring stiffness *k*_d_, *k*_p_, *k*_fm_, just for the rigid body modeling, but in practice they are rotational springs. To this end, in our analysis we consider the DIP, PIP, and MCP joints equivalent to rotational spring loaded joints.

### 3.3. Torsion Spring Modeling

The finger is maintained to its rest position with a torsional spring, that also mechanically implements the passive adduction motion. The stiffness of the torsional spring should be precisely selected. In case of an extremely soft torsional spring, the finger might be sensitive to gravity or to external forces and might also struggle to maintain stable contact points with the object. On the other hand, a highly stiff torsional spring, compared with the elastomer material stiffness, makes the MCP joint to work exclusively as a revolute joint for the finger's flexion motion. Therefore, the actuation mechanism is analyzed to compensate the gravity as a statically balanced mechanism, similarly to Rahman et al. (1995) and Coullet et al. (2009), yet with a torsional spring.

**Figure 4:**
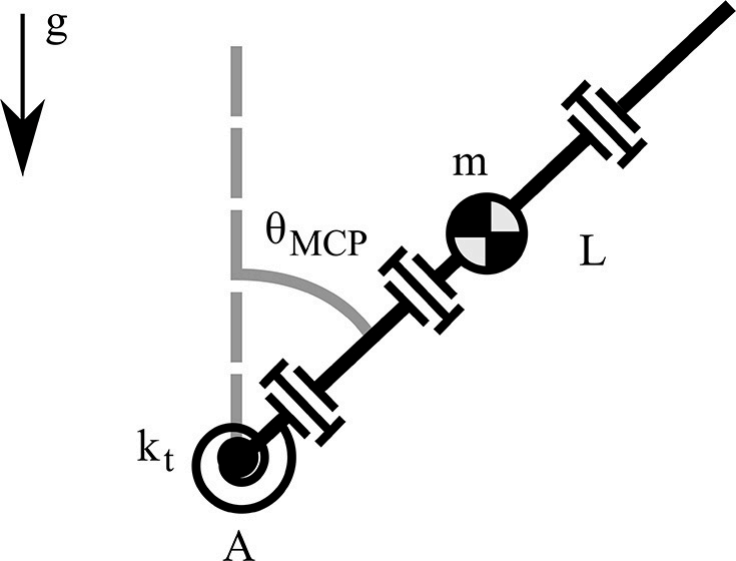
A model of the robotic finger for computing the gravity compensation with a torsional spring.

We seek the greatest lower bound of the torsional spring stiffness that allows the minimum stiffness to compensate for gravity in the worst case scenario. To this end, we consider the rest position, as at this configuration the finger generates the maximum torque about the point A, as depicted in Figure 4. By employing the Euler's equation of motion we obtain,

(1) $$\begin{array}{l} \;\Sigma M_{\text{A}}=I_{\text{A}}{\ddot{\theta }}_{\text{MCP}}\\ \;M_{\text{A}}^{f_{\text{k}}}-M_{\text{A}}^{f_{\text{m}}}=m{\left(\frac{L}{2}\right)}^{2}{\ddot{\theta }}_{\text{MCP}}\\ k_{\text{t}}\theta_{\text{MCP}}-m\text{g}\frac{L}{2}\sin(\theta_{\text{MCP}})=m{\left(\frac{L}{2}\right)}^{2}{\ddot{\theta }}_{\text{MCP}},\\ \; \end{array}$$

where $M_{\text{A}}^{f_{\text{k}}}$ is the restoring moment of the torsional spring about the point A, $M_{\text{A}}^{f_{\text{m}}}$ is the moment of the concentrated mass due to the gravity about the point A, *I*_A_ is the mass moment of inertia of a massless rod about the point A, θ_MCP_ is the abduction angle, ${\ddot{\theta }}_{\text{MCP}}$ is the angular acceleration, *m* is the concentrated mass, g is the gravity acceleration, *L* is the finger length, and *k*_t_ is the stiffness of the torsional spring.

For the statically balanced case the system becomes homogeneous and its angular acceleration is ${\ddot{\theta }}_{\text{MCP}}=0$, so from (1) the torsional spring stiffness results in,

(2) $$\begin{array}{r} k_{\text{t}}\left(\theta_{\text{MCP}}\right)=\frac{m\text{g}L}{2}\mathrm{sinc}\left(\theta_{\text{MCP}}\right), \end{array}$$

where $\mathrm{sinc}\left(\theta_{\text{MCP}}\right)=\frac{\sin\left(\theta_{\text{MCP}}\right)}{\theta_{\text{MCP}}}$ is the cardinal sine function. Next, by considering the small angle approximation, i.e. sin(θ_MCP_) ≈ θ_MCP_, the torsional spring stiffness to compensate gravity in (2) yields,

(3) $$\begin{array}{r} k_{\text{t,min}}<\frac{m\text{g}L}{2}. \end{array}$$

It must be noted that the abduction angle vanishes in (3), making the lower bound of the torsional spring stiffness *k*_t, min_ independent of the abduction angle θ_MCP_ and its initial configuration axis. In addition, by assuming the small angle approximation, the cardinal sine function results its maximum value max{sinc(·)} = 1. Therefore, we obtain the greatest lower bound, inf{*k*_t_} = *k*_t, min_.

The stiffness of the flexure joints is related to the transmitted force to the finger, so the flexure joint stiffness *k*_d_, *k*_p_, *k*_fm_ need to be high. On the other hand, the stiffness of the torsional spring *k*_t_ needs to be stiff enough to compensate for gravity and successfully rebound the finger to its rest position. Therefore, we consider that the stiffness of the flexure joints is larger than the stiffness of the torsional spring, while the torsional spring preserves its minimum computed stiffness to produce appropriate torques τ_d_, τ_p_, τ_fm_> τ _t_. Since we analyze the worst case scenario for the torsional spring, the flexure joints will also compensate for gravity.

### 3.4. Metacarpophalangeal Joint Analysis

Our focus is on the adduction/abduction motion and thus we need to determine the corresponding design characteristics. Since we have already considered the stiffness of the torsional spring *k*_t_ much lower than the stiffness of the flexure joints *k*_d_, *k*_p_, *k*_fm_, we can reduce the model to allow for the mechanism adduction/abduction analysis.

**Figure 5:**
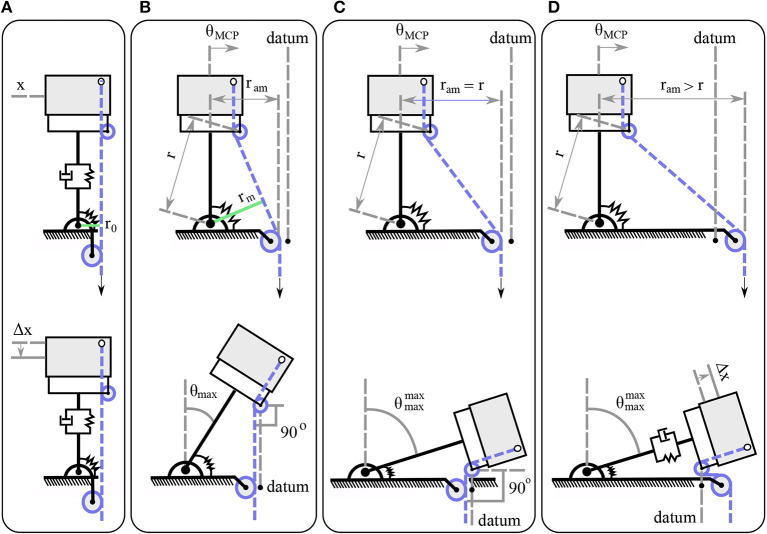
The actuation mechanism in various configurations. Different design choices with respect to the moment arm pulley position, produce various abduction motions. **(A)** The moment arm pulley is perpendicular to the tendon guide pin. **(B)** The moment arm pulley is located at an intermediate position. **(C)** The distance from the joint axis of rotation to the moment arm pulley *r*_am_ matches the radius length *r*. **(D)** The distance from the joint axis of rotation to the moment arm pulley *r*_am_ is higher than the radius length *r*.

The key idea underlying the actuation mechanism is that, by selecting various moment arm pulley positions we will be able to achieve different maximum abduction angles, as presented in Figure 5. That is a dependent motion problem with constraints the tendon length and the datum. The datum is imposed by the moment arm pulley's position. The maximum abduction angle occurs when the finger pulley reaches the datum.

The actuator that is responsible for the adduction/abduction first triggers the abduction until it reaches its highest possible abduction angle and then contributes to the flexion/extension motion, as depicted in the lower part of Figure 5. When the moment arm pulley is by design perpendicular to the tendon guide pin, then the mechanism will trigger only perpendicular motion Δ*x*, as the line of action of the resultant forces will pass through the center of the pulley's axis of rotation, as shown in Figure 5A. In case that we select the position of the moment arm pulley at a horizontal distance *r*_am_, then the mechanism will be abducted until the line of action becomes collinear with the pulley's axis of rotation at θ_max_, as depicted in Figure 5B. Next, for the maximum abduction angle $\theta_{\text{max}}^{\text{max}}$, the moment arm pulley should be placed at a distance *r*_am_ = *r*, as presented in Figure 5C. The last possible choice is to place the moment arm pulley at a distance *r*_am_ > *r* where the finger will first reach its maximum abduction $\theta_{\text{max}}^{\text{max}}$, but then it will be subject to tensile stress with a Δ*x* deformation as shown in Figure 5D. It is to be noted that the moment arm in Figure 5A is relatively small so that the torsional spring and the friction eliminate its effect.

We tackle two problems. First, we specify the design parameters in order to achieve the desired abduction angle θ_max_ in the mechanism. Second, we determine the transferred force to the finger after the friction losses, which are induced by the reconfiguration at the maximum abduction angle θ_max_.

**Figure 6:**
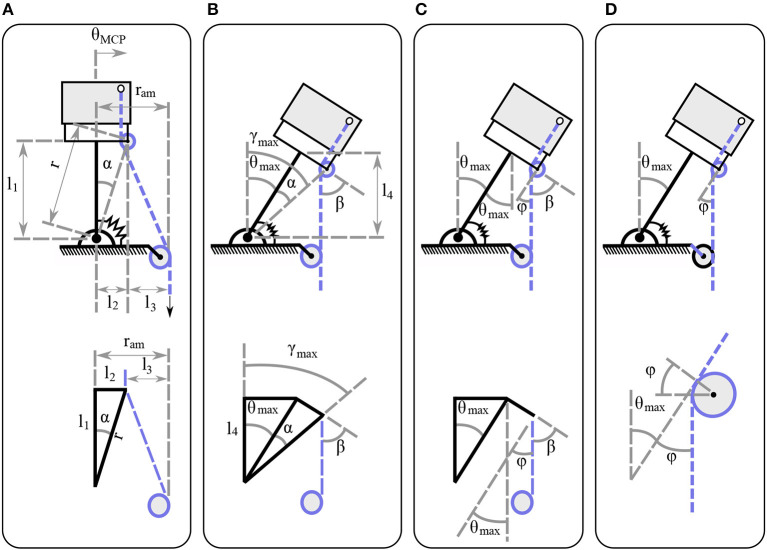
The actuation mechanism model at the initial and at the maximum abduction configuration. **(A)** The mechanism characteristics at the initial configuration for the moment arm pulley position determination. **(B)** The mechanism characteristics at the maximum abduction angle for the moment arm pulley position determination. **(C)** The mechanism characteristics at the maximum abduction angle for the friction losses determination. **(D)** The mechanism characteristics focused on the tendon guide pin at the maximum abduction angle for the friction loss determination.

For the adduction/abduction motion analysis, the actuation mechanism model is depicted in Figure 6. The *l*_1_ is the joint length and *l*_2_ is the finger pulley distance. The actuation mechanism has an internal angle α that is invariant of the actuator displacement and depends only on the mechanism design. The mechanism can achieve abduction angles $\theta_{\text{MCP}}\in \left[-\frac{\pi }{2}+\alpha ,\frac{\pi }{2}-\alpha \right]$ for bidirectional abduction as shown in Figure 5C. Since the analysis deals with clockwise abduction, the mechanism achieves $\theta_{\text{MCP}}\in \left[0,\frac{\pi }{2}-\alpha \right]$, yet the exact same analysis applies for counterclockwise and bidirectional abduction. The angle β is formed by the perpendicular line of the link and the tendon. The length *l*_3_ is the distance from the tendon guide pin to the moment arm pulley. The distance from the abduction joint axis of rotation to the tendon guide pin is illustrated by *r*. As the mechanism performs abduction the distance *r*_am_ remains constant. On the other hand, the perpendicular distance from the abduction joint axis to the guide pin decreases to *l*_4_, when the mechanism arrives at its maximum abduction angle. For the first problem, the variable that imposes the moment arm is also responsible for the maximum abduction angle—that is, the length *l*_3_ at the initial configuration without any actuator displacement. Given the finger design characteristics *l*_1_, *l*_2_ and the desired maximum abduction angle θ_max_, we need to find the distance of the length *l*_3_. At the maximum abduction angle we have θ_max_ = γ_max_−α. Then, from the initial configuration we obtain,

(4) $$\alpha =\arctan\;\left(\frac{l_{2}}{l_{1}}\right).$$

Next, we find that the maximum abduction angle γ_max_ follows,

(5) $$\begin{array}{r} \sin\gamma_{\text{max}}=\sin\left(\theta_{\text{max}}+\alpha \right)=\frac{r_{\text{am}}}{r}. \end{array}$$

Given that *r*_am_ = *l*_2_ + *l*_3_, (5) takes the form of

(6) $$\begin{array}{r} l_{3}=\sin\left(\theta_{\text{max}}+\alpha \right)r-l_{2}. \end{array}$$

Considering that at the initial configuration the length $r=\sqrt{l_{1}^{2}+l_{2}^{2}}$, we express the desired length *l*_3_ exclusively as a function of the maximum abduction angle θ_max_ and the finger design characteristics *l*_1_, *l*_2_ from (6) as follows,

(7) $$l_{3}=\sin\left[\theta_{\text{max}}+\arctan\left(\frac{l_{2}}{l_{1}}\right)\right]\left(\sqrt{l_{1}^{2}+l_{2}^{2}}\right)-l_{2}.$$

For the second problem we consider the Euler-Eytelwein equation $T_{\text{load}}=T_{\text{hold}}{\text{e}}^{\mu \varphi }$, where μ is the friction coefficient. As the finger rotates, the abduction angle θ increases, which results in the reduction of the β angle. Geometrically, we gather that φ and β are complementary, which yields φ = 90−β, as shown in Figure 6C. Therefore, we need to justify the minimum angle φ at the maximum abduction angle θ_max_ to account for the maximum friction exerted forces. The key observation is that the φ angle, at the maximum finger abduction, is geometrically the same as θ_max_, as presented in Figure 6D. Therefore, the available force for the flexion at the maximum abduction position is $T_{\text{hold}}=\frac{T_{\text{load}}}{{\text{e}}^{\mu \theta_{\text{max}}}}$.

### 3.5. Flexure Joint Modeling

In our design, most of the joints employ elastomer materials, i.e., they are flexure joints. To this end, the rotational stiffness of the MCP joint *k*_fm_, of the PIP joint *k*_p_, and of the DIP joint *k*_d_ require further analysis. We employ the smooth curvature model and we provide here a brief description. A detailed analysis of flexure joint stiffness as well as a low-dimensional forward kinematic method can be found in Odhner and Dollar (2012).

The smooth curvature model is an approximation of the Euler-Bernoulli large bending model and utilizes only three parameters. The three parameters are the DoFs of a single link, considering only planar motion. In case of spatial motion the parameters become six, matching their DoFs. To determine the generalized stiffness matrix it is necessary to compute the Hessian of both the internal and external work as follows,

(8) $$\begin{array}{r} {\text{K}}_{\text{flex}}=\nabla \nabla_{\zeta }U-\left(\nabla \nabla_{\zeta }x_{\text{tip}}\right)P_{x}-\left(\nabla \nabla_{\zeta }y_{\text{tip}}\right)P_{y}, \end{array}$$

where ζ = [φ *x y*]^⊺^ ∈ ℝ^3^ is the vector of parameters for planar approximation, *U* is the energy of the flexure, *x*_tip_, *y*_tip_ are the *x*, *y* coordinates of the flexure tip, respectively, and *P*_*x*_, *P*_*y*_ are the applied load at the *x*, *y* directions of the flexure tip respectively. Note that the Hessian of the angle at the flexure tip is eliminated. The energy term in (8) yields,

(9) $$\begin{array}{r} \nabla \nabla_{\zeta }U=\frac{E_{\text{flex}}I_{\text{flex}}}{L_{\text{flex}}}\left[\begin{array}{ccc} 1 & 0 & 0\\ 0 & 1/3 & 0\\ 0 & 0 & 1/5 \end{array}\right], \end{array}$$

where *E*_flex_ is the Young's modulus, *I*_flex_ is the moment of inertia, and *L*_flex_ is the length of the flexure joint. The Young's modulus is assumed to be constant, similar to Stuart et al. (2014). Also, the moment of inertia of a rectangular area is $I_{\text{flex}}=\frac{bh^{3}}{12}$, where *b* is the flexure width and *h* is the flexure thickness.

We consider that the flexure joint is subject to large load and buckling. We account for buckling by employing Euler's critical load equation,

(10) $$\begin{array}{r} P_{\text{cr}}=\frac{\pi^{2}E_{\text{flex}}I_{\text{flex}}}{4L_{\text{flex}}^{2}}. \end{array}$$

Therefore, without any boundary conditions at the flexure tip, i.e., free tip, the (8) from (9) and (10) results in the symmetric global stiffness matrix as

(11) $$\begin{array}{r} {\text{K}}_{\text{flex}}=\frac{E_{\text{flex}}I_{\text{flex}}}{L_{\text{flex}}}\left[\begin{array}{ccc} 1 & 0 & 0\\ 0 & 1/3 & 0\\ 0 & 0 & 1/5 \end{array}\right]+LP_{\text{cr}}\left[\begin{array}{ccc} -1/3 & 1/12 & 1/60\\ 1/12 & -1/30 & 0\\ 1/60 & 0 & -1/210 \end{array}\right]\\ \;=\frac{E_{\text{flex}}I_{\text{flex}}}{L_{\text{flex}}}\left[\begin{array}{ccc} \frac{12-\pi^{2}}{12} & \frac{1}{12} & \frac{1}{60}\\ \frac{1}{12} & \frac{360-\pi^{2}}{360} & 0\\ \frac{1}{60} & 0 & \frac{4200-\pi^{2}}{4200} \end{array}\right]. \end{array}$$

The stiffness matrix **K**_flex_ captures the planar stiffness of the flexure joint associated with the parameter ζ.

**Figure 7:**
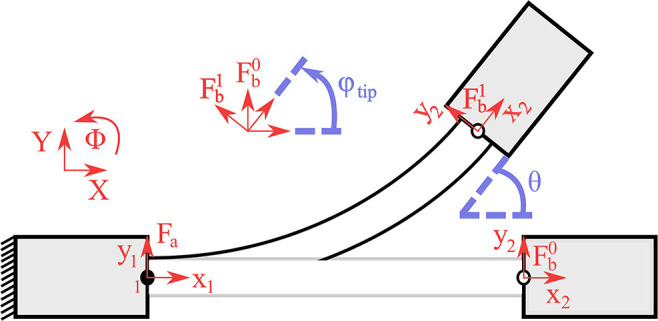
The finger model for spatial motion. **(A)** The abduction θ_MCP_ is performed from the abduction actuator *f*_a_a__. **(B)** The flexion occurs either from the flexion actuator *f*_a_a__ or from the combined motion of both actuators, or from the abduction actuator when θ_MCP_>θ_max_.

Next, we seek to relate the rotational flexure stiffness $k_{\text{flex}}^{\text{rot}}=k_{\text{fm}}=k_{\text{p}}=k_{\text{d}}$ to the global stiffness matrix **K**_flex_. Without loss of generality, we assume straight curvature κ = 0 when then flexure joint is flexed in the free space. The curvature characterizes the planar bending profile of the flexure joint and straight curvature implies that there are neither convex nor concave flexure joint formations. For a comprehensive discussion the reader is referred to Guo and Lee (2013). It must be noted that the smooth curvature model considers only straight curvature. In Figure 7, we present a flexure joint with similar performance to the proposed adaptive finger joints. One side of the joint is fixed to the inertial frame $F_{\text{a}}$ and the other side performs a planar free motion with a body-fixed frame $F_{\text{b}}$ at the flexure tip. It is easy see that the flexure joint configuration matches with the flexure tip rotation, i.e., θ = φ_tip_. This result is analogous to the straight curvature assumption. Thus, the rotational flexure stiffness under large load and buckling takes the form of

(12) $$\begin{array}{r} k_{\text{flex}}^{\text{rot}}=\left[\begin{array}{ccc} 1 & 0 & 0 \end{array}\right]{\text{K}}_{\text{flex}}\left[\begin{array}{c} 1\\ 0\\ 0 \end{array}\right]=\frac{12-\pi^{2}}{12}\frac{E_{\text{flex}}I_{\text{flex}}}{L_{\text{flex}}}\\ \;=0.1775\frac{E_{\text{flex}}I_{\text{flex}}}{L_{\text{flex}}}. \end{array}$$

In case that the flexure joint is only subject to a large load, the smooth curvature model results in rotational flexure stiffness of $k_{\text{flex}}^{\text{rot}}=\frac{E_{\text{flex}}I_{\text{flex}}}{L_{\text{flex}}}$, as in Howell (2012). However, in realistic finger scenarios the flexure joints will be subject to buckling. Since tendon routing systems cannot produce excursively vertical forces to the finger, buckling is inevitable. An illustration of horizontal forces applied to the finger, due to the tendon routing structure, is presented in section 4. To this end, the rotational flexure stiffness is required to be almost five times larger to account for buckling, i.e., multiplied by 0.1775 in (12). Note also that the effect of the flexure joint thickness *h* to the realized stiffness $k_{\text{flex}}^{\text{rot}}$ is proportional to its power of three.

### 3.6. Adaptive Finger Modeling

**Figure 8:**
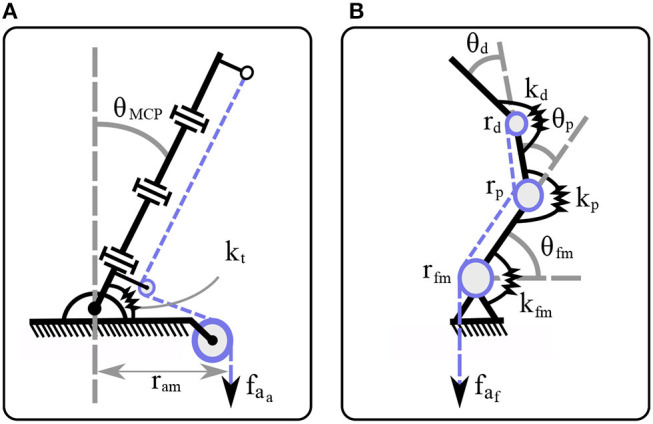
A flexure joint with straight curvature, κ = 0. One link is fixed at inertial frame $F_{\text{a}}$ while the other link performs a planar motion with a body-fixed frame $F_{\text{b}}$. The flexure tip angle, described by the rotation of body-fixed frame $F_{\text{b}}$, is equal to the joint configuration.

The modeling of tendon-driven underactuated fingers has been studied for the planar case in Birglen et al. (2007), Balasubramanian et al. (2012), and Ma and Dollar (2014). However, the proposed mechanism establishes spatial motions and thus a spatial model is required. The kinematics of the coupling finger yield

(13) $$\begin{array}{r} {\text{J}}_{\text{am}}^{⊺}\Delta \theta ={\text{r}}_{\text{a}}\Delta \theta_{\text{am}}, \end{array}$$

where ${\text{J}}_{\text{am}}\in {\mathbb{R}}^{n\times m}$ is the actuation mode Jacobian with *n* denoting the number of DoFs and *m* the number of actuation modes, **θ** ∈ ℝ^*n*^ is the finger configuration, ${\text{r}}_{\text{a}}\in {\mathbb{R}}^{m\times m}$ is the diagonal matrix of the actuator pulley radii values, and $\theta_{\text{am}}\in {\mathbb{R}}^{m}$ is the actuation mode angle vector. Note that we employ (13) to report the kinematics of the finger for every actuation mode. The number of actuators is not equivalent to the number of actuation modes, due to the fact that the actuators can be used both individually and in a combined manner. More specifically, the abduction actuator enforces the MCP abduction, but when both actuators operate then the result is finger flexion. In our analysis, we consider four actuation modes. The first two actuation modes are dedicated to individual actuator operations that reflect either flexion or abduction. The third actuation mode is caused by the abduction actuator which performs abduction until the maximum abduction is achieved, and then triggers the finger flexion. The fourth actuation mode concerns simultaneous motion of the tendon routing systems, which reflects finger flexion. In this way, we can control the adaptive finger in every possible scenario. The actuation mode Jacobian has the form of

(14) $$\begin{array}{r} {\text{J}}_{\text{am}}=\left[\begin{array}{cccc} r_{\text{d}} & 0 & r_{\text{d}} & r_{\text{d}}\\ r_{\text{p}} & 0 & r_{\text{p}} & r_{\text{p}}\\ r_{\text{fm}} & 0 & r_{\text{fm}} & r_{\text{fm}}\\ 0 & r_{\text{am}} & 0 & 0 \end{array}\right]\;, \end{array}$$

where *r*_d_, *r*_p_, *r*_fm_, and *r*_am_ are the pulley radii of the DIP, PIP, flexion MCP, and abduction MCP joints respectively. The finger configuration for every actuation mode is given by $\theta ={\left[\theta_{\text{d}}\theta_{\text{p}}\theta_{\text{fm}}\theta_{\text{MCP}}\right]}^{⊺}$ as presented in Figure 8. For simplicity we consider the actuator pulley values as **r**_a_ = **I**, where **I** is an identity matrix of appropriate dimensions. The actuation mode angle vector is $\theta_{\text{am}}={\left[\theta_{1}\theta_{2\text{a}}\theta_{2\text{f}}\theta_{3}\right]}^{⊺}$, where θ_1_ is the individual operation of actuator 1, θ_3_ is the combined actuators motion, and θ_2a_, and θ_2f_ represent the individual operation of the actuator 2 for abduction and flexion respectively, yielding

(15) $$\theta_{2}=\left\{ \begin{array}{l} \theta_{2\text{a}},\text{if }\theta_{\text{MCP}}\leq \theta_{\text{max}}\\ \theta_{2\text{f}},\text{ otherwise}. \end{array}\right..$$

Therefore, from (13), (14), and (15) we obtain the finger motion for every actuation mode,

(16) $$\begin{array}{r} \Delta \theta_{1}=\Delta \theta_{2\text{f}}=\Delta \theta_{3}=r_{\text{d}}\Delta \theta_{\text{d}}+r_{\text{p}}\Delta \theta_{\text{p}}+r_{\text{fm}}\Delta \theta_{\text{fm}}, \end{array}$$

(17) $$\begin{array}{r} \Delta \theta_{2\text{a}}=r_{\text{am}}\Delta \theta_{\text{MCP}}. \end{array}$$

Next, we present a static balance analysis to obtain the required tendon force for every joint. The static balance equation is given by

(18) $$\begin{array}{r} -\text{K}\Delta \theta +{\text{J}}_{\text{e}}^{⊺}{\text{f}}_{\text{e}}+{\text{J}}_{\text{a}}^{⊺}f_{\text{a}}=0, \end{array}$$

where **K** ∈ ℝ^*n*×*n*^ is the symmetric joint stiffness matrix, **J**_e_ is the disturbance force Jacobian, **f**_e_ is the external forces vector, and $f_{\text{a}}\in {\mathbb{R}}^{+}$ the tendon forces vector. Note that (18) cannot blend various actuation modes and thus the actuation mode Jacobian is reduced to ${\text{J}}_{\text{a}}\in {\mathbb{R}}^{n}$. We assume that there are no external disturbances to the finger, i.e., **f**_e_ = **0**, similarly to Ma and Dollar (2014). The stiffness matrix for the actuation modes that occur in flexion has the form of **K**_1_ = **K**_3_ = **K**_4_ = diag(*k*_d_, *k*_p_, *k*_fm_, 0) and for the abduction actuation mode **K**_2_ = diag(0, 0, 0, *k*_t_). The actuation Jacobians for the flexion actuation mode are **J**_a_1__ = **J**_a_3__ = **J**_a_4__ = [*r*_d_*r*_p_*r*_fm_0], and for the abduction actuation mode **J**_a_2__ = [000*r*_am_]. Therefore, the required tendon force for the flexion *f*_a_f__ and for the abduction *f*_a_a__ without any disturbance yields

(19) $$\begin{array}{r} f_{{\text{a}}_{\text{f}}}=\frac{k_{\text{d}}\Delta \theta_{\text{d}}}{r_{\text{d}}}=\frac{k_{\text{p}}\Delta \theta_{\text{p}}}{r_{\text{p}}}=\frac{k_{\text{fm}}\Delta \theta_{\text{fm}}}{r_{\text{fm}}}, \end{array}$$

(20) $$\begin{array}{r} f_{{\text{a}}_{\text{a}}}=\;\frac{k_{\text{t}}\Delta \theta_{\text{MCP}}}{r_{\text{am}}}. \end{array}$$

For the worst case scenario, which occurs when the finger is flexed at the maximum abduction angle, the tendon force is further reduced to $f_{{\text{a}}_{\text{f}}}^{\text{max}}=\frac{f_{{\text{a}}_{\text{f}}}}{{\text{e}}^{\mu \theta_{\text{max}}}}$ and $f_{{\text{a}}_{\text{a}}}^{\text{max}}=\frac{f_{{\text{a}}_{\text{a}}}}{{\text{e}}^{\mu \theta_{\text{max}}}}$, according to the analysis in section 3.4.

## 4. Developed Finger

In this section, we describe the fabrication process of the anthropomorphic finger, which consists of 3D printing and the Hybrid Deposition Manufacturing (HDM) techniques.

**Figure 9:**
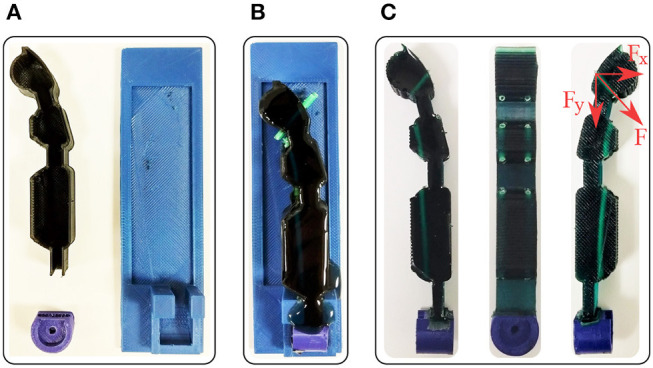
The fabricated adaptive finger and its off-the-self materials. We employed urethane rubber, two 3D printed molds, low friction tubes, and a 3D printed rotating base. **(A)** The reusable mold in blue, the rotating base in purple, and the sacrificial mold in black. **(B)** The elastomer material at the curing phase. **(C)** Side views and front view of the fabricated finger.

The fabrication procedure and the adaptive finger are presented in Figure 9. We employ the HDM technique with two different molds (Ma et al., 2015). More specifically, we use a reusable mold (blue), a rotating base (purple), and a sacrificial mold (black), as presented in Figure 9A. The sacrificial mold has holes to penetrate the low friction tubes (green). Then, the reusable mold accommodates the sacrificial mold, the low friction tubes (green), and the rotating base to prevent elastomer leakage, as depicted in Figure 9B. The rotating base has a special geometry with rounded corners to guarantee a robust interlink of the two bodies (Cutkosky and Kim, 2009). In Figure 9C, the adaptive finger is illustrated in various views. It is evident that the routing of the tendon results in bending forces *F*_*y*_. In case one can produce only vertical forces to the finger *F*_*x*_, the buckling can be neglected.

The finger is monolithic from urethane rubber of shore hardness 80 A (Smooth-On - PMC 780). We utilize the low-friction tubes to reduce the friction in the tendon routing system and 3D printed ABS material for the rotating base to ensure the structure robustness. The weight of the fabricated finger is *m* = 25 g. We exploit the material deformability to enlarge the contact surfaces (Ciocarlie et al., 2005). The design is open source and all the files that are required for the replication and control of the proposed actuation mechanism are freely available through the OpenBionics initiative (Liarokapis et al., 2014). A numerical example on the derivation of the finger characteristics using our analysis is provided in the Appendix (Supplementary Material).

## 5. Results and Experiments

In this section, we assess the effect of friction and we compare the experimental results with the model of the proposed adaptive finger. Then, we evaluate the efficiency of the actuation mechanism and we compute the workspace of the robotic finger. Next, we analyze the grasping forces to investigate the force exertion capabilities of the finger and assure that the joint preserves its position when it is abducted. We also perform a force comparison with a finger at the fully abducted position. Furthermore, we validate the efficacy of the proposed finger design by performing two sets of experiments that include the implementation of various finger configurations and the manipulation of an object. Finally, we fabricate an anthropomorphic, adaptive robot hand based on the proposed mechanism to demonstrate some of its grasping and in-hand manipulation capabilities.

### 5.1. Assessing the Friction of the Tendon Routing

In this subsection, we conduct tendon force experiments to assess the role of friction in the tendon routing system, comparing the required tendon force to bend the finger with the theoretical model results, as discussed in section 3.6.

More precisely, we actuate the finger by applying calibrated hooked weights on the finger tendon and we simultaneously measure the configuration. The finger configuration is obtained with the use of a standard Kinect camera (Microsoft). The joints are tracked with three colored markers, located at the center of the DIP flexure joint, the PIP flexure joint, and the MCP flexure joint. For the lateral motion we place a marker at the fingertip to measure the MCP abduction. Next, we provide the final tendon force required to fully flex the finger. The abduction can be decoupled, and thus studied separately. We conduct 20 trials to derive the mean values and the standard deviations. The computed values are presented in Table 1. The study revealed 37.1% difference for the full finger flexion and 8.1% for the MCP joint abduction when compared with the proposed model. For the flexion of the finger the deviation is higher, as we did not consider friction losses in the tendon routing of the proposed model. However, the required abduction tendon force of the MCP joint closely matches the proposed model value, assuming a friction coefficient of μ = 0.2 in the moment arm pulley. The small standard deviations σ_a_f__ = 0.60 N, and σ_a_a, MCP__ = 0.40 N indicate an acceptable repeatability for the mechanism.

**Table 1:** Required tendon forces.

**Description**	**Theoretical**	**Experimental**	**Standard**
	**tendon forces [*N*]**	**tendon forces [*N*]**	**deviation [*N*]**
Finger F/E	*f*_a_f__ = 8.30	${\bar{f}}_{{\text{a}}_{\text{f}}}=13.20$	σ_a_f__ = 0.60
MCP A/A	*f*_a_a, MCP__ = 1.47	${\bar{f}}_{{\text{a}}_{\text{a,}\text{MCP}}}=1.60$	σ_a_a, MCP__ = 0.40

### 5.2. Finger Postures and Reachable Workspace

In this subsection, we perform finger posture experiments that include an individual finger flexion, an individual finger abduction, a finger flexion at the highest abduction configuration, and a finger abduction at the highest flexion configuration. We also compute the reachable workspace of the proposed adaptive finger.

**Figure 10:**
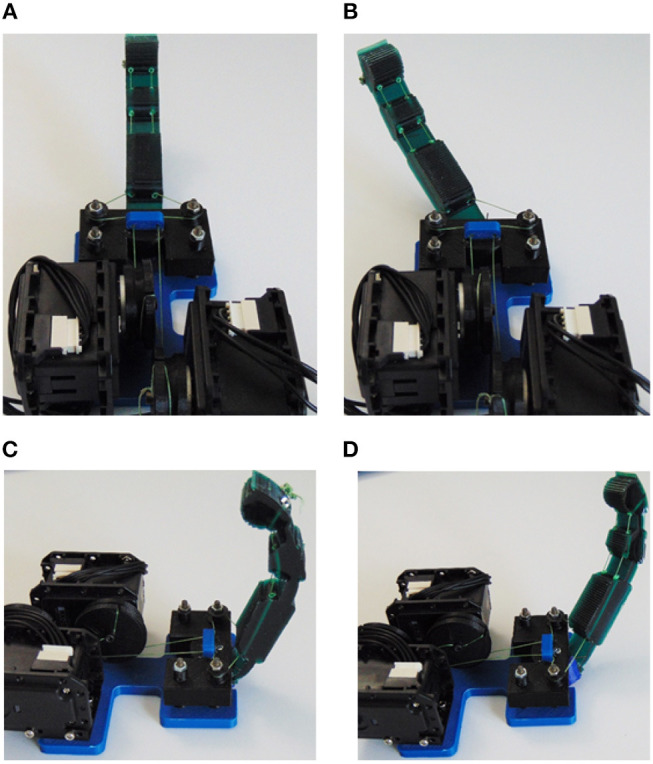
The anthropomorphic finger in various configurations. **(A)** Finger in the neutral (rest) position. **(B)** Finger abduction by using the corresponding tendon-driven system. **(C)** Finger flexion without any abduction. **(D)** Finger flexion in an abducted angle.

**Figure 11:**
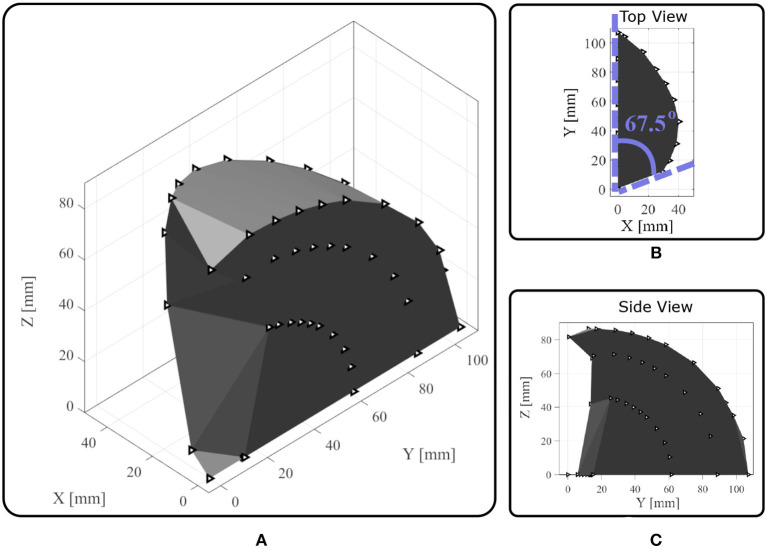
The reachable workspace of an anthropomorphic index finger. The triangles represent the position of the joints through time. **(A)** Perspective view. **(B)** The top view illustrates the finger abduction. **(C)** The side view depicts the finger flexion.

The finger configurations of flexion and abduction are presented in Figure 10. The anthropomorphic index finger equipped with the actuation mechanism is capable of performing adduction/abduction and flexion/extension concurrently. Next, we employ a standard Kinect camera (Microsoft) with three colored markers at the center of each flexure joint, 1 marker at the MCP axis of rotation for the abduction, and 1 marker at the edge of the fingertip. Then, we build the workspace by connecting the 3D points and computing the convex hulls. The anthropomorphic finger was designed to achieve desired maximum abduction angle $\theta_{\text{max}}=67.5^{\text{o}}$. We choose a non-anthropomorphic, extreme abduction angle range to demonstrate the efficacy of the actuation mechanism. In particular, we want to validate our analysis by conducting kinematic experiments with the fabricated finger. In Figure 11, the finger workspace with one side rotation is presented. The maximum angle that was attained by the MCP joint is 67.5^o^, thus our analysis is valid. All the intermediate configurations can be achieved by combing the 2 actuators. Also, the top view in Figure 11B depicts the coupling between the flexion at the extreme abduction angle.

The proposed actuation mechanism is amplifying the workspace compared to finger designs that accomplish only flexion/extension. This workspace extension will allow the execution of dexterous manipulation tasks and facilitate the grasping of large objects.

### 5.3. Force Exertion Capabilities

**Figure 12:**
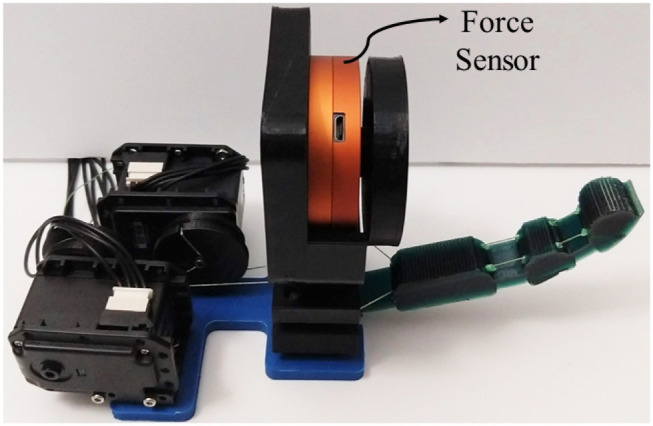
The experimental setup for measuring the finger force exertion capabilities.

We gather the fingertip exerted forces in various configurations of a single digit. To do so we employed the FSE1001 force sensor (Variense), as presented in Figure 12. Next, we measure repeatedly the exerted forces that occurred for only flexion by employing both actuators. Similarly, we measure the fingertip forces in fully abducted configuration by employing again both actuators. The experimental procedure is adopted from the finger strength measure protocol proposed by Falco et al. (2015). That is a kinetic measure of the maximum force that a robotic finger can impose on its environment. In addition, we evaluated the force exertion capabilities during abduction.

**Figure 13:**
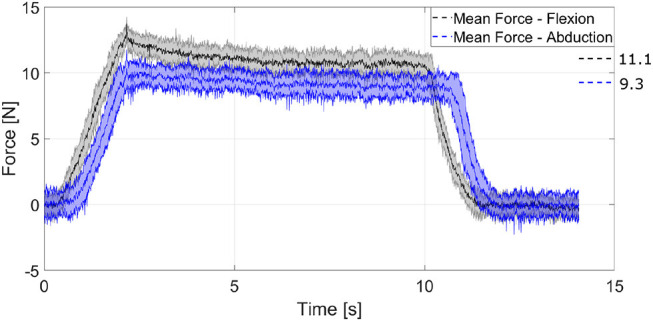
A comparison of the fingertip exerted forces in two different configurations. The performance of the finger is quite similar at every trial. The finger exerts similar forces even at the fully abducted position. The momentum of the finger during contact generates the overshoot region. The long decay after the overshoot represents the strain energy stored in the elastomer material of the flexure joints, which results in a reconfiguration of the finger toward an elastic equilibrium, as presented in Liarokapis and Dollar (2018).

We acquire the fingers forces from 20 trials. The comparison of the fingertip exerted forces in two configurations is shown in Figure 13. The overall mean exerted forces are illustrated on the right side with dashed line. The solid line represents the mean value at each time, while the shadowed area depicts the standard deviation. The black-gray colored area depicts the finger forces with only flexion and the blue-light colored area depicts the finger forces at flexion in a fully abducted position. The reported overall mean value for flexion only (for the quasi-static case, i.e., by ignoring the dynamic contact forces) is 11.1 N. The standard deviation reveals that the performance of the finger is similar at every trial. The actuation mechanism not only maintains its position from the maximum abducted configuration while flexed, but it also reports a force of 9.3 N. As the finger is abducted, the achievable finger force is reduced, because of various friction losses, yet they remain significantly high. Therefore, the proposed actuation mechanism allows competitive finger exerted forces even at the fully abducted position, which facilitates the execution of robust grasping actions.

### 5.4. Grasping and Manipulation Capabilities

For the grasping and manipulation experiments we used a cylindrical object. The object was fabricated from ABS 3D printed material (stiff), with diameter *D* = 25 mm and length *h* = 50 mm.

**Figure 14:**
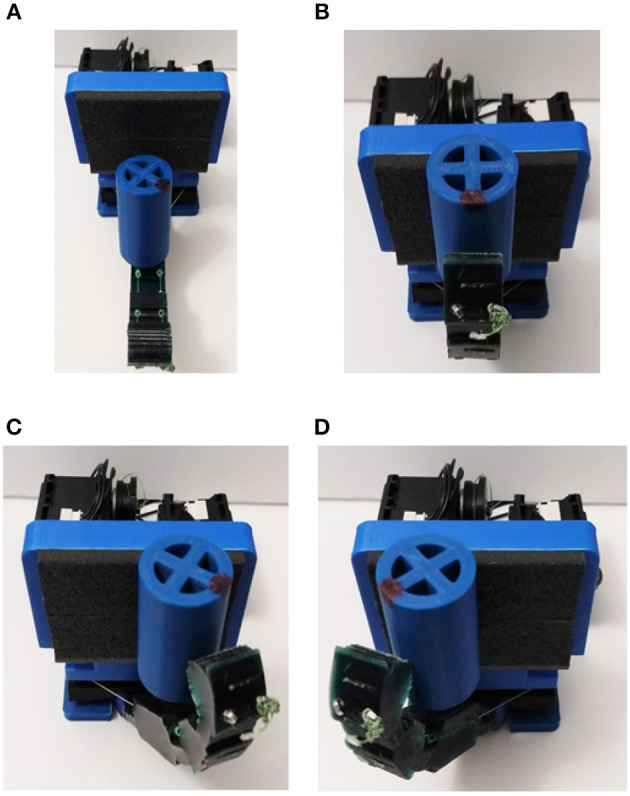
The anthropomorphic finger performing a manipulation task. **(A)** The adaptive finger with the cylindrical object. **(B)** Initial grasping position. **(C)** Rolling counterclockwise 45^o^. **(D)** Rolling clockwise 90^o^.

The grasping and manipulation experiments are depicted in Figure 14. First, the finger performed a robust grasping action. Then, the finger rolls the object bidirectionally from 0^o^ to −45^o^, from −45^o^ to 45^o^, and from −45^o^ to 0^o^. The rolling did not cause a significant object slip as it successfully returns at its initial position, which is indicated by a black mark on the object, as demonstrated in the attached Video S1. This experiment reveals the grasping and manipulation capabilities of a single bidirectional adaptive finger equipped with the proposed actuation mechanism.

### 5.5. Anthropomorphic Robot Hand Paradigm

As an illustrative example, we consider an anthropomorphic, adaptive robot hand that is based on the proposed actuation mechanism (Kontoudis, 2018). The hand is comprised of five fingers, two parallel lever-based differential mechanisms, and four tendon routing systems actuated by four motors, having a ratio of actuators per finger of 0.8. The design of all fingers uses the proposed actuation mechanism. More specifically, for the index finger we make use of the right-side anchor point to achieve clockwise abduction, for the ring and pinky fingers we make use of left-side anchor points to achieve counterclockwise abduction, and for the thumb we employ both-side anchor points to achieve bi-directional abduction. The four fingers are actuated by two actuators through the two parallel differential mechanisms. Since the thumb is the most important finger of the hand, we dedicate to its motion two actuators.

**Figure 15:**
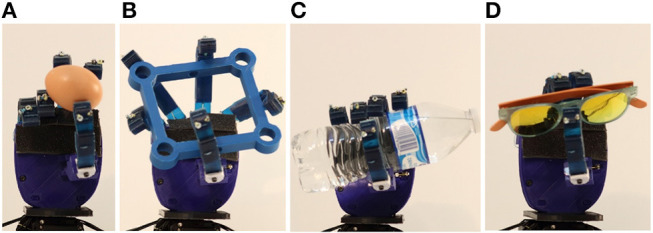
Grasping of various everyday-life objects **(A)** Raw egg. **(B)** Rectangular object. **(C)** Bottle of water. **(D)** Pair of sunglasses.

We perform various grasping experiments with everyday-life objects, as presented in Figure 15. Particularly, we perform a tripod, circular, precision grasp for the raw egg and the pair of sunglasses, a disk, circular precision grasp for the rectangular object, and a power grasp for the bottle of water, as shown in the attached Video S1. The underactuation and the structural compliance of the proposed adaptive mechanism enforce grasping and handling of delicate objects, e.g., the raw egg and the pair of sunglasses, without breaking them. Also, the abduction of the four fingers enhances the grasping of large objects, e.g., the rectangular object.

**Figure 16:**
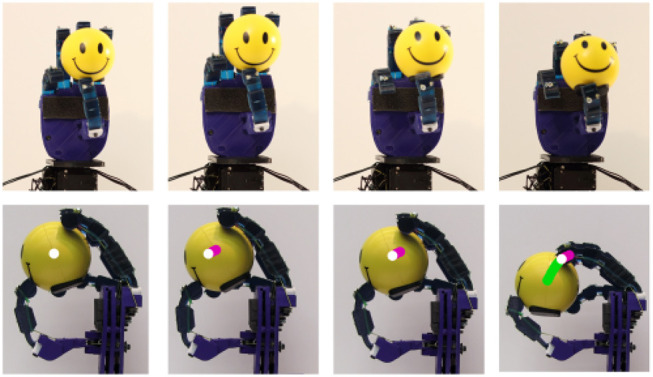
An equilibrium point manipulation experiment executed with the examined robot hand that is equipped with the proposed mechanism. The robot hand successfully grasps a spherical object and then performs rolling of the object, which results in an equilibrium point motion.

An example of an equilibrium point manipulation task executed with a small plastic ball is depicted in Figure 16. The robot hand rolls the object, yet the rolling is not simple and results also in equilibrium point manipulation motion, as shown in the attached Video S1. The initial equilibrium point position is illustrated in white. The first equilibrium point motion is depicted in pink as shown in the second column of Figure 16. The major equilibrium point motion is illustrated in green in the last column of Figure 16. All these experiments validate the efficiency of the proposed actuation mechanism.

## 6. Discussion

In this section, we present limitations of the actuation mechanism which are imposed by specific design choices. We also discuss future directions that may improve the performance and reliability of the proposed mechanism.

### 6.1. Design Limitations

Certain limitations arise from the underactuated nature of the adaptive actuation mechanism. In particular, we employ 2 actuators to control 4 joints, i.e., an actuators-to-joints ratio of 0.5. This ratio can be further reduced with the use of differential mechanisms (Birglen et al., 2007). For example, one may use 2 actuators to achieve flexion/extension and adduction/abduction of *n*-fingers with a parallel structure of whiffletree. However, even for the simplest case of one finger, which we discussed in this paper, the individual control of each joint is not possible. That is a common disadvantage of every underactuated mechanism. Although this compromise reduces the dexterous manipulation capabilities, in this work we have provided some dexterous manipulation examples (e.g., object rolling and equilibrium point manipulation).

Another limitation that originates from underactuation is due to the passive extension of the finger. A passive extension mechanism does not allow for the active control of finger extension or the development of forces in the opposite direction of flexion. There are applications where the robot hand is assigned to grasp or manipulate objects in cluttered environments. In such cases, the robot hand needs to relocate objects without grasping, but by pushing them aside to clear the field of view. For this scenario, the capability to actively control the extension of fingers is important and thus, for these cases, variable stiffness, fully actuated devices (Haas et al., 2018) may be more applicable.

### 6.2. Future Directions in Design and Modeling

The goal of the experimental procedure was to validate the design and analysis of the proposed actuation mechanism. The repeatability of the current mechanism was not designed to ensure industrial standards. For instance, after some trials we observed that the tendon routing system was relaxed, i.e., backlash appeared. This occurred because the termination of the tendon routing system was poorly considered. To address this problem, a termination mechanism that allows for fine tendon termination end adjustment (Gerez and Liarokapis, 2018) may be utilized.

An important factor for the calculation of the actuator size is the friction of the tendon routing system. In this work, we discussed the friction that is introduced by the moment arm pulley of the actuation mechanism. We also computed the required tendon force, yet without considering other friction losses. To this end, we used an oversized actuator to compensate for potential non-modeled friction losses of the tendon routing system. However, for an accurate actuator selection an analytical friction model should be developed. Moreover, the friction model has to precisely determine the position of each contact of the tendon routing system. The importance of these contact points is two-fold—first, to analytically model friction and, second, to determine the torque distribution from the tendon routing system to the finger. More specifically, a torque is generated at every contact point of the tendon routing system that is perpendicular to the finger. An initial discussion is provided in Gerez et al. (2019). By employing such models we will be able to precisely compute the forward kinematics (FK) of the finger by just knowing the actuator displacement. Although the authors of the smooth curvature model provide a methodology for the FK computation with a flexure joint, the accurate tip position of adaptive fingers with multiple flexure joints depends on the contact points of the tendon routing system. It must be noted that the assessment of the role of friction in the tendon routing system was discussed in section 5.1.

Another approach of computing the FK is to sensorize the flexure joints with stretchable sensors (Polygerinos et al., 2017). In fact the later approach may be more valuable, as feedback of the flexure joint configuration will be available even when the robot hand interacts with either the environment or the objects. Also, the joint configuration may be employed by proprioception techniques (Vàsquez and Perdereau, 2017) to estimate the object configuration and/or environmental characteristics.

## 7. Conclusions and Future Work

This paper proposed a tendon-driven actuation mechanism for adaptive robot hands. More precisely, we developed anthropomorphic, adaptive fingers that are equipped with an MCP joint capable of implementing flexion/extension and adduction/abduction concurrently. We presented the joint's specifications and we proposed a modeling framework that compensates for gravity. We also performed a mechanism analysis that derives the appropriate parameters for the implementation of various abduction configurations. A finger model was discussed that predicts the finger motion, computes the required tendon force for every actuation mode, and derives the stiffness value for each flexure joint. The exerted force results show a force range between 9.3 N and 11.1 N for the two extreme configurations by exploiting the torque of both actuators. Moreover, the workspace has increased significantly, indicating an enhancement in the overall system dexterity. Next, we validated the actuation mechanism's performance by providing experimental paradigms conducted with the developed anthropomorphic, adaptive thumb finger. The finger achieves adduction/abduction and flexion/extension concurrently, which allows the execution of both robust grasping and dexterous manipulation tasks. Furthermore, the finger is able to execute both robust grasping tasks and dexterous manipulation tasks with insignificant slip. An anthropomorphic adaptive robot hand is used as a paradigm to illustrate the mechanism capabilities. The hand maintains an actuators-to-fingers ratio of below 1, achieving various grasping tasks, finger interdigitation, and equilibrium point manipulation. The latter is considered as one of the most complex dexterous, in-hand manipulation tasks.

## Author Contributions

GK significantly contributed to the modeling and the analysis of the actuation mechanism, the modeling of the finger, the fabrication of the adaptive finger, the design and fabrication of the adaptive robot hand, the execution of the experiments, the analysis of the results, and the writing of the manuscript. ML significantly contributed to the modeling and the analysis of the finger, the analysis of the results, and the writing of the manuscript. KV and TF significantly contributed to the the analysis of the results and the writing of the manuscript.

### Conflict of Interest Statement

The authors declare that the research was conducted in the absence of any commercial or financial relationships that could be construed as a potential conflict of interest.

## Footnotes

^1^The four fingers refer to the index, middle, ring, and pinky.

## Supplementary Material

Video S1 The video presents the proposed finger equipped with the actuation mechanism. In particular, we show the finger at its extreme configurations and we present a rolling manipulation experiment of a cylindrical object. Alongside the experiments, each actuator's contribution is depicted. The video also illustrates the experiments of an anthropomorphic adaptive robot hand equipped with the proposed actuation mechanism, which enhances its grasping and dexterous in-hand manipulation capabilities.

Presentation S1 The Appendix provides a numerical example of the adaptive finger characteristics.
