# Supplementary material for: An Adaptive Actuation Mechanism for Anthropomorphic Robot Hands
Source: Front Robot AI. 2019 Jul 5;6:47. doi: 10.3389/frobt.2019.00047 (PMC7805973; doi:10.3389/frobt.2019.00047)
Supplement: Presentation S1 — The Appendix provides a numerical example of the adaptive finger characteristics. [file Presentation_1.pdf]

Appendix

In this section, we provide a detailed example for the derivation of the anthropomorphic finger characteristics. We derive the dimensions of the adaptive finger based on anthropometric data, Buchholz et al. (1992). Since the finger length and finger breadth are predetermined, the only free parameter that we can vary to achieve different rotational flexure stiffness values, is the flexure joint thickness h. The design parameters that affect the finger's motion are the moment arm pulley position, the elastomer material stiffness of the flexure joints, and the torsional spring stiffness. The ideal bending profile of the finger that depends on the stiffness ratio of the flexure joints is selected using the anthropomorphism index proposed by Liarokapis et al. (2013), and the maximum desired flexion angle range, Kapandji (1974). Given that the phalanges' dimensions dominate the mass calculation, we approximate the mass of the finger using the material density. Moreover, certain limitations in space are imposed by the tendon routing system. The parameters required for the computation of: i) the rotational flexure stiffness, ii) the distance of the moment arm pulley, and iii) the required tendon force to actuate the finger, are listed in Tables S1, S2, S3. In Table S1, the dimensions and the approximated mass of the whole finger are presented. The finger structure is anthropomorphic and its parameters were computed for hand length HL$=185$ mm and hand breadth HB$=90$ mm, as discussed in Kontoudis et al. (2015). Table S2 shows the radii, the angle ranges, the tendon distances, and the lengths of the flexure joints that perform flexion/extension. In Table S3, we list the elastomer material properties as given by the manufacturer (Smooth-On).

First, we compute the radius of the moment arm pulley for a desired abduction. According to our analysis in Subsection 3.2, for desired maximum abduction angle $\theta_{\text{max}} = 67.5^{\text{o}}$, MCP flexure joint length $L_{\text{flex,m}} = l_1 = 7.00$ mm, and tendon distance $l_2 = 5.10$ mm, the required distance from the abduction joint axis to the moment arm pulley from (7) results to, $r_{\text{am}} = l_2 + l_3 = 5.10 + 3.32 = 8.42$ mm, as presented in Table S4. We choose a non-anthropomorphic, extreme abduction angle range to demonstrate the efficacy of the actuation mechanism. In particular, we want to validate our analysis by conducting kinematic experiments with the fabricated finger. We show in Subsection 5.2 that the finger achieves the desired maximum abduction angle of 67.5^{o}.

Next, we compute analytically the rotational flexure stiffness of the MCP joint, k_{fm} and we list the other two rotational flexure stiffness of the PIP joint, k_{p} and the DIP joint, k_{d}, as the procedure is identical. Let us employ (3) to obtain the minimum torsional spring stiffness that mechanically rebounds the finger to its initial position and concurrently compensates gravity,

$$k_{\text{t,min}} = \frac{0.025 \times 9.81 \times 0.088}{2} = 0.0105 \frac{\text{N.m}}{\text{rad}}. \tag{S1}$$

We utilize the required flexure joint thickness to produce slightly larger rotational flexure stiffness to the MCP joint. We consider flexure joint thickness, $h_{\text{fm}} = 5.00$ mm and flexure joint length, $L_{\text{flex,m}} = l_1 = 7.00$ mm. The resulted stiffness is computed by (12) as,

$$k_{\text{fm}} = 0.1775 \frac{2.76 \times 10^6 \times \frac{16.20 \times 10^{-3} \times (5.00 \times 10^{-3})^3}{12}}{7.00 \times 10^{-3}} = 0.0120 \frac{\text{N.m}}{\text{rad}}. \tag{S2}$$

Table S1. Robotic Finger Characteristics

Description	*Parameter*	*Value*
Length	L [mm]	88.00
Width	b [mm]	16.20
Phalange Thickness	h [mm]	15.00
Estimated Mass	$\hat{m}$ [g]	25.25

Table S2. Flexure Joint Characteristics for Flexion

Joints	*Radii* [mm]	*Angle Ranges* [deg]	*Tendon Distance* [mm]	*Lengths* [mm]
DIP F/E	$r_{\text{d}} = 10.00$	$\Delta\theta_{\text{d}} = 20$ to 90	$3{*}l_2 = 5.10$	$L_{\text{flex,d}} = 6.00$
PIP F/E	$r_{\text{p}} = 10.00$	$\Delta\theta_{\text{p}} = 0$ to 100		$L_{\text{flex,p}} = 6.00$
MCP F/E	$r_{\text{fm}} = 12.50$	$\Delta\theta_{\text{fm}} = 0$ to 80		$L_{\text{flex,m}} = 7.00$

Table S3. Urethane Rubber (Smooth-On PMC 780) Properties

Description	*Parameter*	*Value*
Density	ρ [kg/m^3]	1020
Young's Modulus (100%)	E [MPa]	2.76
Shore Hardness	S [A]	80

Table S4. MCP Joint Characteristics for Abduction

Description	*Parameter*	*Value*
Desired Maximum Abduction	θ_{max} [deg]	67.50
Tendon Distance	l_2 [mm]	5.10
Length	$L_{\text{flex,m}} = l_1$ [mm]	7.00
Computed Radius	$r_{\text{am}} = l_2 + l_3$ [mm]	8.42

Table S5. Rotational Joint Stiffness and Tendon Force

Joints	*Thickness* [mm]	*Stiffness* [N.m/rad]	*Tendon Forces* [N]
DIP F/E	$h_{\text{d}} = 6.00$	$k_{\text{d}} = 0.0235$	$f_{\text{af,DIP}} = 2.87$
PIP F/E	$h_{\text{p}} = 6.00$	$k_{\text{p}} = 0.0235$	$f_{\text{af,PIP}} = 4.10$
MCP F/E	$h_{\text{fm}} = 5.00$	$k_{\text{fm}} = 0.0120$	$f_{\text{af,MCP}} = 1.33$
MCP A/A	-	$k_{\text{t}} = 0.0105$	$f_{\text{a}_{\text{a,MCP}}} = 1.47$

Similarly, the rotational flexure stiffness of the PIP and DIP joints are, $k_{\text{p}} = k_{\text{d}} = 0.0235\frac{\text{N.m}}{\text{rad}}$, with flexure joint thickness, $h_{\text{p}} = h_{\text{d}} = 6.00$ mm and flexure joint length, $L_{\text{flex,d}} = L_{\text{flex,p}} = 6.00$ mm, as shown in Table S5.

Lastly, we compute the required tendon force for each actuator by utilizing (19), (20). We consider the desired flexion ranges for every joint. The total tendon force required for fully bending the finger yields, $f_{\text{af}} = f_{\text{af,DIP}} + f_{\text{af,PIP}} + f_{\text{af,MCP}} = 8.30$ N. Likewise, the required tendon force for the maximum abduction of the finger is, $f_{\text{a}_{\text{a}}} = f_{\text{a}_{\text{aMCP}}} = 1.47$ N. We equip the robotic finger with 2 Dynamixel RX-28 servo motors with torque $T_{\text{m}} = 2.8$ Nm at 12V and outer shaft diameter $D_{\text{m}} = 2.50$ mm for the flexion/extension and

adduction/abduction. Then, we place a pulley to the outer shaft with diameter $D_\mathrm{p} = 50.00$ mm. The resulting tendon force of each actuator is $f_\mathrm{a} = 112$ N. Note that we use motors with significantly higher torque to guarantee robustness of grasping.

REFERENCES

Buchholz, B., Armstrong, T. J., and Goldstein, S. A. (1992). Anthropometric data for describing the kinematics of the human hand. *Ergonomics* 35, 261–273

Kapandji, I. (1974). *Physiology of the joints: Upper limb*, vol. 1 (Churchill Livingstone Edinburgh), 6 edn.

Kontoudis, G. P., Liarokapis, M. V., Zisimatos, A. G., Mavrogiannis, C. I., and Kyriakopoulos, K. J. (2015). Open-source, anthropomorphic, underactuated robot hands with a selectively lockable differential mechanism: Towards affordable prostheses. In *IEEE/RSJ International Conference on Intelligent Robots and Systems*. 5857–5862

Liarokapis, M. V., Artemiadis, P. K., and Kyriakopoulos, K. J. (2013). Quantifying anthropomorphism of robot hands. In *IEEE International Conference on Robotics and Automation*. 2041–2046
